# Examination of Throats of Children

**Published:** 1897-10

**Authors:** 


### EXAMINATION OF THROATS OF CHILDREN.

The *New York Medical Review* calls attention to a simple and effective means of throat examination, which should be more generally known.

It is well known that many children have a dread of the doctor's visit—especially should the visit be made because of throat disease. The fears are increased if a spoon or tongue depressor is thrust down into the throat without ceremony. All of this may be overcome by a method used by me for the past twelve years, which can be successfully practiced in nearly every patient over three years of age. It consists in simply teaching the child to use the index finger of either hand, thrust back along the tongue as near the base as possible, with the injunction to open the mouth wide and press down the tongue. In this way can be secured, after one or two attempts, a perfect view of the tonsils, and in many instances even of the epiglottis and the adjacent folds.

The reason why this is preferred is based on, first, the fact that a child, or even an adult, does not fear any injury from his own finger; second, his own effort will not provoke emesis or straining, as a trial will convince the reader; third, there is no danger of contamination by a dirty spoon or depressor, and no possibility of auto-infection, and, finally, the fingers are always at hand. This plan, of course, is impracticable in the moribund and in infants, but at least 95 per cent. of all instances of acute and chronic disease of the throat or of foreign bodies can be more successfully examined by it than by any other method.

